# Research trends in drama therapy: a bibliometric analysis based on Scopus

**DOI:** 10.3389/fpsyg.2023.1327656

**Published:** 2023-12-21

**Authors:** Žanete Korde, Sanita Šuriņa, Kristīne Mārtinsone

**Affiliations:** Department of Health Psychology and Pedagogy, Riga Stradins University, Riga, Latvia

**Keywords:** drama therapy, dramatherapy, creative arts therapies, health care, research trends, scientific publications, bibliometric analysis, Scopus

## Abstract

**Aim:**

As drama therapy has become increasingly important in healthcare, the number of scientific publications has increased, complicating the orientation in the research field. Therefore, the aim of this study is to provide a comprehensive overview of research trends in drama therapy, assessing the impact factors of publications and analyzing the research structure.

**Methods:**

Three hundred and ninety-five scientific articles indexed in Scopus were analyzed without time, geographic and language restrictions using bibliometric analysis methods - performance analysis, citation analysis, and science mapping. Data processing was performed using MS Excel, VOSviewer and Biblioshiny software.

**Results:**

The work of the pioneers of drama therapy continues to be actively cited, influencing the development of drama therapy. Today’s leading researchers are increasingly engaging in collaborative research, working across disciplines and contributing to scientific progress. The hot topic of research is older adults and mental health. The diversity of terminology and the large volume of unpublished research point to the need for consolidation. Altmetric analysis would optimize the use of scientific information, promoting innovative research.

**Conclusion:**

This is the first study using the Scopus database to perform an extensive bibliometric analysis of research articles on drama therapy applying Biblioshiny and VOSviewer software. The results of the analysis reflect the evolution of the field from its historical roots to academic maturity, highlighting its current dynamic growth and the trend of drama therapy to establish itself as an interdisciplinary field in the healthcare system. This work serves as a valuable resource for the scientific community, professionals, students, and librarians in the field, helping to optimize the use of scientific resources in drama therapy and contributing to its future development.

## Introduction

Digital globalization (Henriques, 2019), together with the rapidly growing volume of scientific publications and new data analysis tools (Liu et al., 2023), is impacting scientific research worldwide. In this context, the lack of systematization in drama therapy research has been recognized (Fernández-Aguayo and Pino-Juste, 2018; Constien and Junker, 2023), leading to difficulties in navigating the scientific literature. To address this problem, an extensive bibliometric analysis of the research structure is needed (Pessin et al., 2023). This research is essential to make effective use of the scientific resources in drama therapy and to develop this area of research further. It facilitates the synthesis of new ideas for future research, simplifies the orientation of drama therapy professionals to scientific work and offers additional sources of literature for drama therapy students and librarians in educational institutions. It also provides insight for other interested parties on the possibilities of collaboration in the field of drama therapy research.

Nowadays, drama therapy is the deliberate use of drama and/or theatrical processes to achieve therapeutic goals (Emunah, 2019; North American Drama Therapy Association, 2021), namely symptom relief, emotional, cognitive, and physical integration, and personal growth (SANATA, 2020). In many parts of the world, drama therapy is an integrated mental health and human services profession (American Art Therapy Association, 2022; Davies and Clift, 2022) and is applied in a variety of settings across the lifespan (North American Drama Therapy Association, 2021). The World Health Organization and the Lancet global research series clearly point to the growing importance of the arts, including drama therapy, in health and its positive impact on emotional and social well-being (Jensen and Bonde, 2018; Fancourt and Finn, 2019).

Drama therapy has its roots in different traditions and theoretical approaches (SANATA, 2020). From a historical perspective, only a few points of emphasis are discussed here. Its origins lie in the development of Jacob Levi Moreno’s psychodrama method. Moreno was a pioneer of group psychotherapy and the founder of psychodrama, but later psychodrama and drama therapy developed differently (Orkibi et al., 2023). The emergence of theoretical concepts of drama therapy began in the middle of the last century. Books began to be published in both Europe and the United States, and the first periodicals describing the content of drama therapists’ practice were produced. The first professional associations were founded (The British Association of Dramatherapists, 2020) and the first educational programs were implemented until, in 1999, drama therapy was included among the health professions in the United Kingdom under the supervision of the Health Professions Council (Karkou and Sanderson, 2006). In the United States, drama therapy has also been established as a separate profession (North American Drama Therapy Association, 2021). In the Dutch legislation, drama therapy has the status of creative therapy (Federatie Vaktherapeutische Beroepen, 2023), while in Latvia and Israel drama therapy is one of the specializations within the profession of art therapy, which also includes visual-plastic, music, dance, movement therapies (Latvian Dramatherapy Association, 2023), bibliotherapy and psychodrama (Israel Association of Art Therapy, 2019). In many parts of the world, drama therapy is used as a modality in other professional practices. Although drama therapy has a different status in different countries, there are efforts to coordinate its development in order to achieve comparable education and professional practice (Johnson et al., 2012; Emunah, 2019). The founding of the European Federation of Drama Therapy in 2013 and the establishment of the World Alliance of Drama Therapy in 2017 evidenced these efforts (The European Federation of Dramatherapy, 2017; The World Alliance of Dramatherapy, 2017).

With the increasing number of scientific publications (Liu et al., 2023), following research in one’s field of specialization becomes increasingly difficult (Fernández-Aguayo and Pino-Juste, 2018; Feniger-Schaal and Orkibi, 2020; Constien and Junker, 2023), as researchers must undertake extensive data analysis and interpretation (Pessin et al., 2023). The first initiatives in this regard have been taken by professional bodies, including the British Association for Drama Therapists (BADth), which was founded in 1977 (Dokter and Winn, 2009), the North American Drama Therapy Association (NADTA), which was founded in 1979 (Armstrong et al., 2019), and the Federation of Vocational Therapy Associations (FVB), which was founded in 2006 (Federatie Vaktherapeutische Beroepen, 2023). These organizations have collected publications in the field of drama therapy through online databases (Frydman et al., 2022) and have concluded that the professional practice of drama therapy is characterized by low professional visibility, relatively reduced remuneration and lack of institutional support (Armstrong et al., 2019). Systematic reviews and meta-analyses that have already been conducted have highlighted empirical findings and expanded the theoretical base of drama therapy. Scientific research has become increasingly important around the world in recent years, driven by the growth of big data, new measurement approaches and a growing range of empirical methods. These tools provide opportunities to test conceptual frameworks of science, uncover factors that influence scientific productivity, predict key scientific outcomes and design policies that facilitate future scientific progress better (Liu et al., 2023). A suitable method for exploring large volumes of scientific data is bibliometric analysis, which is used to explore the characteristics, structure, relationships, patterns, and current and future trends of scientific disciplines quantitatively and qualitatively (Soosaraei et al., 2018).

To the best of our knowledge, bibliometric analysis of research in the field of drama therapy has been carried out twice. First, in 2018, Professor Sara Fernández-Aguayo and her colleagues conducted a review of programs using drama therapy or theatre as a therapeutic method, with the aim of systematizing methods, techniques, and outcomes through content analysis (Fernández-Aguayo and Pino-Juste, 2018). The study included data from 1974 to 2017, and the results showed that drama therapy is used more in health care, while theatre is used more in education. The study found that in terms of publication prevalence, the most research in drama therapy was published in the journal The Arts in Psychotherapy. The results showed that many authors have published only one article. It was also concluded that there is a lack of a common conceptual and terminological systematization (Fernández-Aguayo and Pino-Juste, 2018). A broader picture of drama therapy research was then provided by a bibliometric analysis of the literature from the database of the Institute for Research and Development in the Arts Therapies at the Nürtingen-Geislingen University, analyzed by Tobias Constien and Professor Johannes Junker (Constien and Junker, 2023). The study included data from 2000 to 2021 and assessed several quantitative indicators. The study revealed a significant increase in the number of publications as well as a trend towards more collaboration between researchers in the field of drama therapy. Articles are mainly from the United Kingdom and the United States and are more concentrated in specialist journals. Moreover, like the review of drama therapy programs by Fernández-Aguayo and Pino-Juste (2018), this analysis pointed to a lack of systematization of studies, which affects the availability of publications. The need for a citation analysis was highlighted, to identify the most influential authors, institutions, countries, journals and publications in the field of drama therapy. The need for a conceptual structure analysis to facilitate the systematisation of terminology is also noted.

Digital globalization is radically reshaping the boundaries between groups and nations, offering new opportunities for collaboration, as also highlighted by the World Health Organization’s call for interdisciplinary collaboration (Fancourt and Finn, 2019). This is also the case at the International Conference on Drama Therapy, organized by the European Federation for Drama Therapy in collaboration with the World Alliance for Drama Therapy, on 5 May 2023 in Amersfoort, the Netherlands, where Dr. Fancourt presented a keynote address to the European Drama Therapy Alliance on 5 May 2023 in Amersfoort, the Netherlands. Nisha Sajnani, founder of the World Alliance for Drama Therapy, called on researchers to encourage collaboration across borders, cultures, and languages (The European Federation of Dramatherapy, 2017). This call highlights the need to explore collaboration patterns between drama therapy researchers and to extend previous bibliometric analysis to all languages. The previous analysis cited as a limitation the focus on only English language publications, excluding studies of local relevance in other languages (Constien and Junker, 2023). Previously they did not explore the research structure of the field by analyzing the links between different research components, e.g., authors, countries, topics (Donthu et al., 2021; Mukherjee et al., 2022). Finally, the historical roots of the research have not yet been explored but a holistic bibliometric analysis of historical drama therapy publications can contribute to understanding the intellectual structure of the research field by facilitating academics and practitioners to navigate the scientific literature (Kokol et al., 2021).

To summarize the above knowledge gap, the aim of the study was to provide an overview of research trends in the field of drama therapy by assessing scientific performance, identifying publication impact indicators and describing the social, intellectual and conceptual structure of research. This includes a time-unlimited study of the worldwide distribution of science based on a bibliometric analysis of the components of scientific publications indexed in the Scopus database, including languages, fields, journals, organisations, authors, countries, citations, references and keywords. Bibliometric analysis thus provides a holistic view of the research field, identifies and characterizes research gaps, and synthesizes new ideas for future research (Donthu et al., 2020, 2021; Mukherjee et al., 2022).

To measure scientific performance, the following research question (RQ1) is asked: What are the productivity indicators of research in the field of drama therapy indexed in Scopus, including the evolution of scientific performance over time, in which research areas and in which languages?

To identify impact indicators, the following research question (RQ2) is asked: Which are the most influential authors, institutions, countries, and journals in the field of drama therapy that have published scientific publications indexed in Scopus, and which are the most popular?

To assess the social structure of the research field of drama therapy, the following research question (RQ3) was posed: Which drama therapy researchers and countries have collaborated most frequently in the studies indexed in Scopus?

In order to describe the intellectual structure of the research field of drama therapy, the following research question (RQ4) is posed: Which are the documents that researchers in drama therapy studies indexed in the Scopus database referred to most frequently?

In order to describe the conceptual structure of the drama therapy research field, the following research question (RQ5) is posed: which are the most frequent keywords in the studies indexed in the Scopus database, is there a thematic correlation between them, and are there current research themes?

## Materials and methods

### Methods and techniques

There are three types of bibliometric indicators: quantitative indicators, which measure the productivity of a particular researcher; qualitative indicators, which measure the quality of a researcher’s work; and structural indicators, which analyse the relationship between publications or their components, such as authors and countries (Durieux and Gevenois, 2010).

Bibliometric analysis mainly uses two main methods: performance analysis, which measures productivity and impact, and science mapping, which describes the relationship between publications or their components (Donthu et al., 2021). There are numerous indicators for performance analysis, the most important of which are the number of publications and citations per year or per research component, where publications are a measure of productivity and citations are a measure of impact. Other indicators, such as citations per publication and the h-index, combine both citations and publications to measure the performance or contribution of research components (Donthu et al., 2021). As standard practice in bibliometric analysis (Mukherjee et al., 2022), this study conducted an analysis of scientific performance, which provided insight into the research landscape in the field of drama therapy. Science mapping explores the relationships between the components of a research area (Donthu et al., 2021) and this study used the techniques of science mapping - citation analysis, co-citation analysis, co-authorship analysis and keyword analysis. Citation analysis is based on the assumption that citations reveal links between publications and identify influential publications in a research field. Co-citation analysis assumes that publications that are frequently cited together are thematically similar and reveal the intellectual structure of a research field and its underlying themes. Co-authorship analysis explores the interactions between authors and their affiliations and describes the social structure of the research field that influences its development. Keyword analysis suggests that words that frequently appear together have a thematic relationship with each other and can be used to reveal topical issues and predict future research topics. These techniques, combined with network analysis, show the structure of the research field.

**Figure 1:**
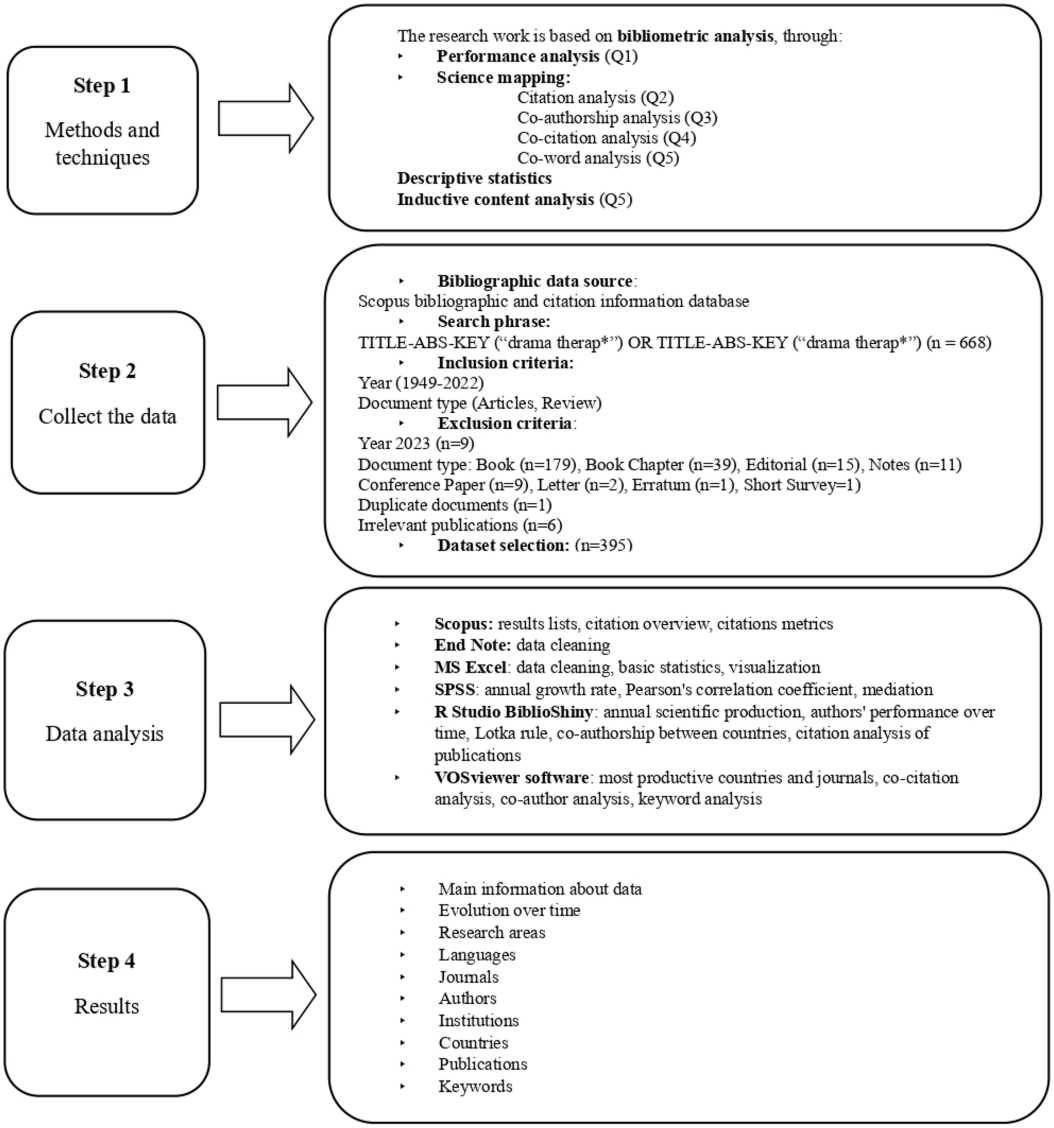
Process of research methodology.

The data were presented in graphs, tables, charts, visualized in figures and descriptive statistics were used to analyze the data. Qualitative analysis of the keywords was carried out using inductive contingency analysis. Figure 1, step 1 shows the methods and techniques used to answer the research questions.

### Collect the data

Metadata for scientific publications is stored in online bibliographic and citation information databases such as Web of Science (Clarivate Analytics) and Scopus (Elsevier). The decision to choose one of the databases was made after identifying differences in indexation between the databases and a large number of duplicates (*n* = 250) of studies in the field of drama therapy in the above-mentioned databases. More papers were identified in Scopus (*n* = 668) compared to Web of Science (*n* = 503), so the data used in this study were extracted from the Elsevier company’s Scopus database. Access to the databases was provided through the Riga Stradins University online library portal in July 2023.

The bibliographic search was conducted using the keyword string (TITLE-ABS-KEY (“drama therap*”) OR TITLE-ABS-KEY (“dramatherap*”)), selecting articles whose titles, abstracts or keywords mentioned drama therapy or its derivatives. Keyword selection was based on the key academic terms identified in the initial literature review that were relevant to the field of study. To include as many data sources as possible, a wildcard “*” was used to allow for variable keyword endings. For example, “therap*” included both “therapy” and “therapist.” Language was not restricted, as it was found that several studies were conducted in countries where the main language was not English (*n* = 42). In the dataset, 668 records were obtained from the Scopus database. The main items considered suitable for the bibliometric study were scientific articles and reviews published in peer-reviewed scientific journals (Okubo, 1997; Glänzel, 2003). The start date of the search was not provided, allowing the database to identify the first scientific publication on drama therapy in 1949.

Books (*n* = 179), book chapters (*n* = 39), editorials (*n* = 15), notes (*n* = 11), conference papers (*n* = 9), letters (*n* = 2), corrections (*n* = 1) and short surveys (*n* = 1) were excluded. To ensure accurate calculations for the annual cut-off, studies published in 2023 were excluded. To identify duplicates, the search result was exported to the reference management tool EndNote (Clarivate Analytics), where two identical publications were identified, of which the one published later was excluded (Lothane, 2011). Given the historical development of drama therapy and its different status in different countries, all studies that mentioned drama therapy were included, regardless of the extent and context in which it was mentioned. This allowed drama therapy to be studied with a comprehensive approach to the diverse context of the field’s development, as well as its integration into other professional fields. The manual review of documents excluded studies that were not related to drama therapy (*n* = 6; Johnston, 2009; Blencowe et al., 2018; Kawano et al., 2018; Suh et al., 2021; Davico et al., 2022). This phase of the study was triangulated twice - the first time after reading the study titles, keywords, and abstracts, but the second time after reading the content of the controversial studies. After this evaluation, 395 documents were selected for inclusion in the subsequent analysis. Step 2 of Figure 1 illustrates the process of building the dataset, indicating the database, the search phrase for inclusion and exclusion criteria.

### Data analysis

An authorized profile was created in the Scopus database, where a selection of the publications included in the study was stored. For further analysis, the data were exported to MS Excel, where they were structured and categorized by several fields: author, ID, title, DOI, year of publication, journal information (ISSN, ISBN), affiliated institution, abstract, author keywords, indexed keywords, references, language, and document type. Data cleaning was used to correct identified errors, such as author surnames in different languages and different numbers of initials, as well as mismatches between journal and institution names. Step 3 of Figure 1 shows the tools and programs used, revealing how they were used in the research method to analyze and visualize the data.

The bibliometric analysis used the results lists available in the Scopus database, which included documents by type, subject area, affiliation, and languages. A citation overview was extracted from the Scopus database, separating data with and without self-citations. The Scopus database only allowed the selection of citation overviews from 1970 onwards and for a maximum of 15 years at a time. Therefore, the data were downloaded and exported to MS Excel in 4 parts, covering the periods 1970–1984, 1985–1999, 2000–2014 and 2015–2022. The data were then merged and sorted according to the research questions. Hirsch index values and journal citation metrics were also extracted from the Scopus database.

In MS Excel, according to the research questions, the required publication information items were manually listed, cleaned, analyzed, and visualized, and bibliometric performance indicators were compiled. Those indicators that could not be obtained with Scopus were analyzed with VOSviewer (version 1.6.19) and Biblioshiny software, which are frequently used in research (Arruda et al., 2022; Srivastava and Srivastava, 2022; Sun et al., 2022) and suitable for those without technical knowledge in programming. The software is freely available for download and installation on any computer (Ahmi, 2022). To ensure data accuracy, the data obtained in VOSviewer and BiblioShiny were compared with the information items in MS Excel.

RStudio software is designed for big data and offers several analysis packages (Firdaus et al., 2019). In this study, the RStudioBibliometrix software was used, which is specifically suited for bibliometric analysis (Aria and Cuccurullo, 2017). The selected dataset from Scopus was exported and processed using Bibliometrix, which allowed researchers to visualize the annual scientific production, to analyze the authors’ performance over time, to investigate the Lotka rule and to assess co-authorship between countries, as well as to perform citation analysis of publications.

VOSviewer is a widely used software tool for visualizing and constructing bibliometric network maps, developed in 2009 by van Eck and Waltman of Leiden University (van Eck and Waltman, 2010). Three different visualization maps can be generated with VOSviewer, including network, density, or coverage visualization maps with different meanings. In these maps, a node represents an element such as country, institution, journal, document, author, or keyword. Links between nodes represent relationships between elements. Several factors, including the number of publications, citations or frequency of repetition determine the size of an element. Using VOSviewer software, the most productive countries and journals were identified, a co-citation analysis and a co-author and keyword analysis were carried out, complemented by a network analysis.

IBM SPSS Stastics (Statistical Package for the Social Sciences, version 29.0) was used to analyze the annual percentage growth rate, Pearson’s product–moment correlation coefficient and mediation.

## Results

### Main information about data

To answer the research question (RQ1) on the productivity of studies conducted in the field of drama therapy and indexed in the Scopus database, a bibliometric analysis method - performance analysis, was used following the recommendations of the bibliometric analysis guidelines (Donthu et al., 2021). The main outcomes of performance analysis include publication-related indicators, citation-related indicators, and publication and citation-related indicators, which are summarized in Table 1. Information on publications, type, research years and Hirsch index was extracted from the Scopus database results lists. MS Excel software was used to compile data on contributing authors, sole-author and co-author publications, active research years, average publication age, number of citations and number of citations per year. The total number of references was determined using VOSviewer. This is a clear overview to present the overall picture of research results in the field of drama therapy.

**Table 1:** Main metrics for performance analysis.

Description	Abbreviations and formulas	Results
*Publication-related metrics*		
Total publications	TP	395
Article		353
Review		42
Number of contributing authors	NCA	755
Sole-authored publications	SA	187
Co-authored publications	CA	208
Number of active years of publication	NAY	50
Productivity per active year of publication	PAY = TP/NAY	7.9
Mean age of publication		12.26
*Citation-related metrics*		
Total citations	TC	2966
Average citations	AC = TC/TP	7.51
Mean number of citations per year per doc		0.95
References		14098
*Citation and publication related metrics*		
Collaboration index	CI = (NCP/TP)/TP	0.002
Collaboration coefficient	CC = 1 − (TP/NCA)	0.48
Number of cited publications	NCP	287
Proportion of cited publications	PCP=NCP/TP	0.97
Citations per cited publication	CCP = TC/NCP	10.34
h-index	h	28

### Evolution over time

To answer the research question (RQ1) on the productivity of studies conducted in the field of drama therapy and indexed in the Scopus database, the evolution of scientific performance over time was analyzed. The analysis was conducted using Biblioshiny software, IBM SPSS Statistics, and descriptive statistical methods including correlation analysis and moderation analysis were applied.

**Figure 2:**
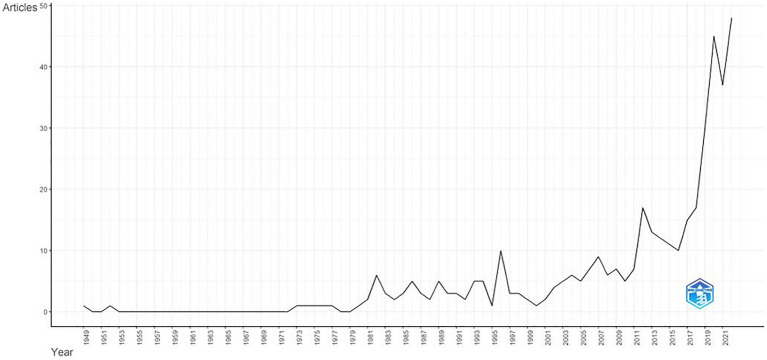
Evolution of the frequency of publications in drama therapy over time.

The study analyzed a period of 74 years, starting from the first Scopus-indexed publication in the field of drama therapy in 1949 in the Bulletin of the Menninger Clinic (Bikales, 1949). Figure 2 shows a graph that visually represents the dynamics of drama therapy publications over these years. Analyzing this period, we found that between 1949 and 1980, the average number of publications per year ranged from 0 to 1. During this period, no work was published for 24 years. However, from 1981 onwards, there was an increase in the number of publications.

The median year in which 50% of the total number of publications was achieved was 2016. The most productive year was 2022, when 47 studies were published. The annual growth rate of scientific output was 5.45%, with an average of 7.9 publications per year.

The Spearman rank correlation coefficient indicates a significant and positive association between year of publication and number of articles published, *r* (73) = 0.854, 95% CI [0.75, 0.92], *p* < 0.001. This relationship suggests that the number of articles published annually in the field of drama therapy is increasing at a constant rate.

### Research areas

To answer research question (RQ1), the research areas in which research in the field of drama therapy has been conducted and indexed in Scopus were identified. Information on the research areas was obtained by analyzing the lists of results available in the Scopus database. More than half of the drama therapy research is concentrated in the medical field (28.42%), psychology (26.04%) and health care (23.42%), but the fields of arts and humanities (10.23%) and social sccienes (6.06%) are also important research contexts.

### Languages

To answer the research question (RQ1) on the productivity of studies in the field of drama therapy indexed in Scopus, we analyzed the number of studies published and in which languages. Language usage data were extracted using the results lists available in the Scopus database. Publications in 17 different languages were identified, with English being the leading language with 363 (91.90%) publications. This was followed by French with 9 (2.28%) publications, and German in third place with 7 (1.77%) publications. Also identified were 4 publications in Spanish, 3 in Czech and two each in Greek and Russian. In addition, there were 10 publications, one in each of the other languages, and 5 publications in 2 languages.

### Journals

To answer the research question (RQ1), what are the productivity indicators of studies in the field of drama therapy indexed in the Scopus database, the prevalence of publications in journals was determined using the VOSviewer software. Using MS Excel, journal titles, number of publications and number of citations were initially compared and refined. A journal with different titles was identified, i.e., Arts in Psychotherapy and The Arts in Psychotherapy, but both journals had the same ISSN (01974556). When analyzing the publication data, it became clear that the two titles referred to the same journal, so they were combined and the results were summed. The papers analyzed in this study were published in 160 different journals, of which 124 (78%) contained only one study in the field of drama therapy. The highest number of publications was found in Arts in Psychotherapy, which is an international journal for all arts therapy specialties and for psychotherapy professionals and researchers. This was followed by Drama Therapy Review, published in collaboration with the North American Drama Therapy Association. In third place was Frontiers in Psychology, a prominent international journal that publishes strictly peer-reviewed research in all branches of psychology. These three journals accounted for almost half (48.61%) of all research publications.

In response to the research question (RQ2) on the most influential journals in the field of drama therapy publishing scientific papers indexed in Scopus, several Scopus citation metrics, including SCImago Journal Rank (SJR), Source Normalized Impact per Paper (SNIP) and CiteScore, were used to assess the quality, visibility, and impact of a journal in this specific field. In 2022, Frontiers in Psychology had the highest Scopus citation metrics, with a SCImago Journal Rank (SJR) of 0.891, Source Normalized Impact per Paper (SNIP) of 1.422 and CiteScore of 4.5. Table 2 provides more information on journals that published at least three studies in the field of drama therapy and most highly results in each category are in bold.

**Table 2:** Journals with more than three publications.

Rank	Journal	FP	TP	TC	CS	SJR	SNIP
1	Arts in Psychotherapy (The Arts in Psychotherapy)	1996 (1982)	**128**	**1598**	2.9	0.46	0.826
2	Drama Therapy Review	2019	53	123	1.6	0.37	0.384
3	Frontiers in Psychology	2018	11	162	**4.5**	**0.89**	**1.422**
4	Body Movement and Dance in Psychotherapy	2009	4	16	1.5	0.31	0.752
5	British Journal of Learning Disabilities	2013	4	32	1.8	0.41	0.95
6	Journal of Creativity in Mental Health	2007	4	35	1.4	0.25	0.573

FP, first publication; TP, total publications; TC, total citations; CS, CiteScore 2022; SJR, SCImago Journal Rank 2022; SNIP, Source Normalized Impact per Paper 2022.

### Authors

To answer the research question (Q1) on the productivity of studies in the field of drama therapy indexed in Scopus, the most productive authors were identified using Biblioshiny. The bibliometric data of the authors - surnames, initials, identification numbers (IDs) - were reviewed in MS Excel and authors with the same surname and one of the initials but with different IDs were identified - Smith M.E. and Smith M., Wood L., and Wood L.L. Having established the identity of the authors, we found that M.E. and Smith M. were different persons, but that Wood L.L. and Wood L. are the same person and the different identification numbers are an error in the Scopus database, so the bibliometric data of these authors were merged. Authors with different surnames in different languages or different numbers of initials but the same identification number (Author(s) ID) have been identified as different entities - Bielańska A. and Bielanska A., Mondolfi Miguel M.L. and Mondolfi M.L., Savage M.D. and Savage M., Sayre D.N. and Sayre D., Shamir O.Y. and Shamir O. With the same ID number but with Moye K.D. and Moye K. listed as two separate co-authors, these data were also merged. One paper is authored by the organization (Chartered Society of Physiotherapy, 1990). After data cleaning, 972 author signatures were identified with a range of one to 18 signatures per document.

**Figure 3:**
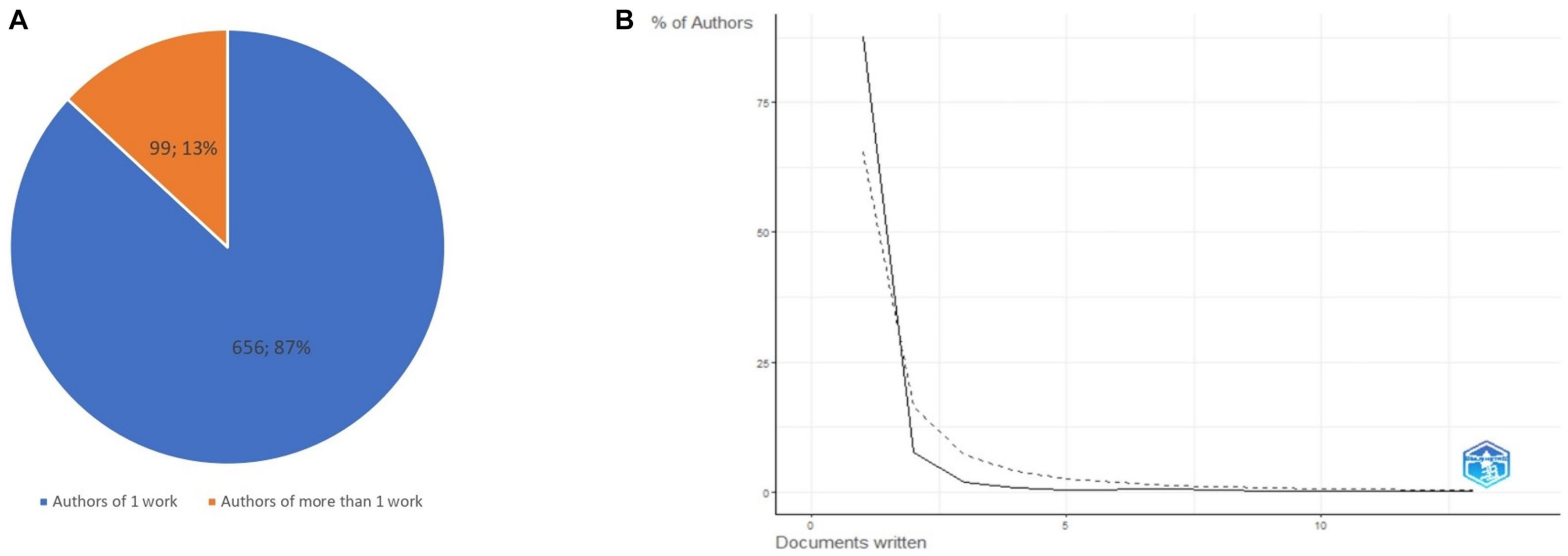
Author’s productivity in drama therapy studies. **(A)** The proportion of the number of authors with single or multiple papers. **(B)** Authors Productivity through Lotka’s Law.

Figure 3A shows a chart of the authors’ productivity indicators created in MS Excel. Of the 755 authors, 656 (87%) authors published only one study and 99 (13%) authors published more than one study. The relationship between the number of authors and the number of contributions (articles) of each author is defined by Lotka’s law (Lotka, 1926). Alfred Lotka found that the number of published papers is not evenly distributed among authors and that productivity tends to concentrate in a limited number of researchers (Okubo, 1997; Glänzel, 2003). This is expressed mathematically by the following formula: A(*n*) = A (1)*n*2A (*n*) = A (1)*n*2, where A(*n*) is the number of authors who have published n papers and A1 is the number of authors who have published one paper. Figure 3B shows a graph created in Biblioshiny comparing the percentage of authors in the dataset of this study based on their number of published works with Lotka’s predictions. A full counting method was applied, where each co-author of a publication was assigned one point. The area up to the solid line represents the empirical or observed data in this study, while the area up to the dotted line represents the theoretical relationships.

To answer the research question (RQ2) about the most influential authors in the field of drama therapy, the Biblioshiny application was used and, in addition to the number of publications, the authors’ citations were analyzed. In line with the Leiden Manifesto, which emphasizes the evaluation of research good practice, a set of indicators was considered (Hicks et al., 2015). Productivity and citability were assessed using different indicators, and are shown in Table 3. The top 10 results for each type of analysis are in bold. Productivity was assessed for each author in two ways: full, where each co-author receives one point, and fractional, where each co-author receives 1/n points for an article with n authors (Rousseau et al., 2018). The most prolific author was Robert Landy (New York University, United States), who published 13 papers over a 26-year period from 1982 to 2007. The authors’ total number of citations and the number of citations without self-citations were analyzed. The most cited author with 234 citations (194 citations without self-citations) was David Johnson from Post Traumatic Stress Center, New Haven, United States. Combining the number of publications and the citation rate, three types of local index values for the authors’ scientific performance were determined: the H, G, and M index. The Authors’ Local Index value describes the authors’ performance in this sample of studies, excluding publications not related to drama therapy. The Hirsch index focuses on the dispersion of citations, the G index considers the total number of citations of all articles, and the M index allows a more objective comparison between researchers whose length of career differs significantly (Bornmann et al., 2008). The most influential new authors were Powell Joanne (Edge Hill University, United Kingdom) and Zoe Moula (UCL Institute of Education, United Kingdom) with the highest M index value. The results of the analysis show that Hod Orkibi (University of Haifa, Israel) had a high value for all indicators.

**Table 3:** Most influential authors.

Rank	Author	FP	Affiliation^*^	Country	NP	NPF	h	g	m	TC	CES
1	Johnson D.R.	1982	Post Traumatic Stress Center	United States	**9**	**7.2**	**9**	**9**	0.21	**234**	**194**
2	Landy R.J.	1982	New York University	United States	**13**	**11.1**	**8**	**13**	0.19	**176**	**160**
3	Orkibi H.	2010	University of Haifa	Israel	**10**	**6.18**	**8**	**10**	**0.57**	**217**	**120**
4	Pendzik S.	2003	Hebrew University of Jerusalem	Israel	**10**	**6.42**	**7**	**10**	0.33	**142**	**107**
5	Sajnani N.	2013	New York University	United States	5	1.65	**5**	5	**0.46**	**170**	**119**
6	Feniger-Schaal R.	2013	University of Haifa	Israel	**7**	**3.2**	**5**	**7**	**0.46**	86	53
7	Karkou V.	2012	Edge Hill University	United Kingdom	**7**	2.02	**5**	**7**	0.42	87	44
8	Frydman J.S.	2016	Lesley University	United States	**8**	**4.53**	**5**	**7**	**0.63**	60	33
9	Mayor C.	2012	Lesley University	United States	**7**	**4.63**	**5**	**7**	0.42	61	30
10	Keisari S.	2017	University of Haifa	Israel	**8**	**3.75**	**5**	**8**	**0.71**	79	28
11	Rousseau C.	2005	School of Medicine	Canada	4	0.95	4	4	0.21	**145**	**115**
12	Koch S.C.	2015	Faculty of Fine Arts and Music	Australia	4	0.66	4	4	0.44	**131**	**98**
13	Jones P.	2008	IOE	United Kingdom	7	**6.17**	4	**6**	0.25	41	38
14	Palgi Y.	2017	University of Haifa	Israel	4	1.25	4	4	**0.57**	61	24
15	Baker F.A.	2018	Faculty of Fine Arts and Music	Australia	3	0.44	3	3	**0.50**	**140**	**105**
16	Gauthier M.F.	2005	Université McGill	Canada	3	0.45	3	3	0.16	**116**	**91**
17	Moula Z.	2020	UCL Institute of Education	United Kingdom	3	0.92	3	3	**0.75**	22	5
18	Powell J.	2020	Edge Hill University	United Kingdom	3	0.92	3	3	**0.75**	22	5
19	Kantor J.	2019	Univerzita Palackého v Olomouci	Czech Republic	5	1.2	3	3	**0.60**	15	5
20	Kaimal G.	2019	Drexel University	United States	2	0.19	2	2	0.40	**93**	**64**
21	Wood L.L.	2018	Lesley University	United States	**10**	**2.03**	2	4	0.33	19	9

^*^Information on affiliation bases on the most recent publication. FP, first publication; NP, number of publications; NPF, number of publications fractionalized; h, h-index; g, g-index; m, m-index; TC, total citations; CES, citations exclude self-citations.

**Figure 4:**
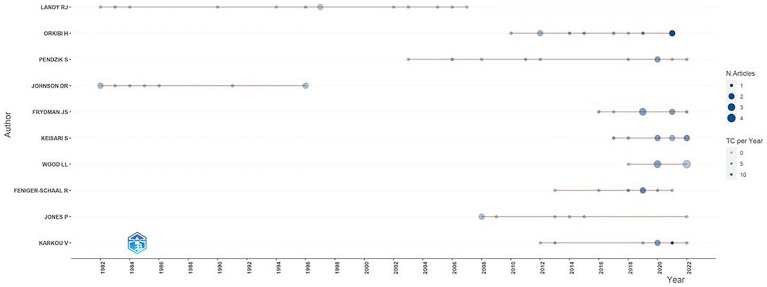
Author’s production over time.

To provide information on the performance of drama therapy authors over time, timelines of the scientific performance of the 10 most productive authors in the field of drama therapy from 1949 to 2022 have been visualized using the Biblioshiny application. In the graph of Figure 4, each point represents the year in which the corresponding author published at least one paper. The size of the dots corresponds to the number of papers published in that year, and the intensity of the color indicates how often the author has been cited. The graphic shows the publication periods of drama therapy pioneers Robert Lundy and David Reid Johnson from 1982 to 1996. Noteworthy is also the period of Susan Pendzik’s scientific activity from 2003 to the present. The schedule also shows the most active contemporary authors, including the highly cited Hod Orkibi.

**Figure 5:**
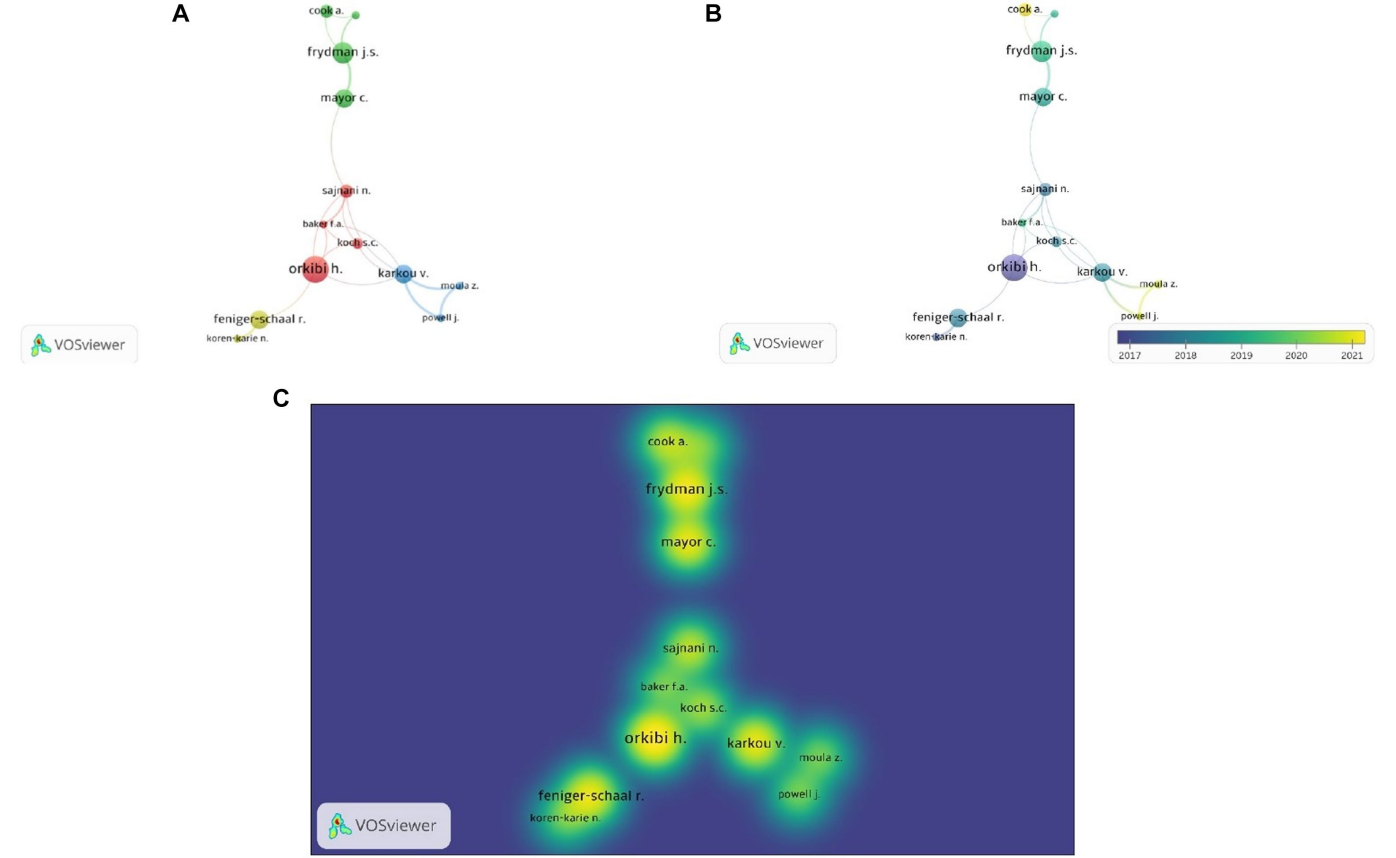
Authors’ collaborations. **(A)** Network Visualization. **(B)** Overlay Visualization. **(C)** Density Visualization.

Using MS Excel, it was determined that of the 755 authors in the study, 145 (19.21%) were solo authors (authors who wrote the articles alone) and 610 (80.79%) were collective authors. The average number of authors per publication was 1.92 between 1949 and 2016, 2.81 between 2017 and 2019, and 3.31 between 2020 and 2022. To answer the research question (RQ3) concerning the most frequent collaborations between researchers in the field of drama therapy, a co-authorship analysis was performed using the VOSviewer application. Figure 5A shows a visualization of the co-authorship network. Collaborating authors were grouped into clusters and marked with different colors, and the links between them indicate collaboration. If there were multiple collaborations between two authors, the links were merged into one. The red cluster (cluster 1) included Baker F.A. (Australia), Koch S.C. (Australia), Orkibi H. (Israel), Sajnani N. (United States). The green cluster (cluster 2) included Armstrong C.R. (Canada), Cook A. (United States), Frydman J.S. (United States), Mayor C. (United States). The blue cluster (cluster 3) included the following authors: Karkou V. (United States), Moula Z. (United Kingdom), Powell J.L.L. (United Kingdom). The yellow cluster (cluster 4) included Feniger-Schaal R. (Israel), Koren-Karie N. (Israel). Figure 5B shows the trends of the authors’ collaborations over time and Figure 5C shows the intensity of the authors’ scientific activity in terms of number of publications and citations, as well as number of collaborations, with red indicating higher rates.

### Institutions

**Figure 6:**
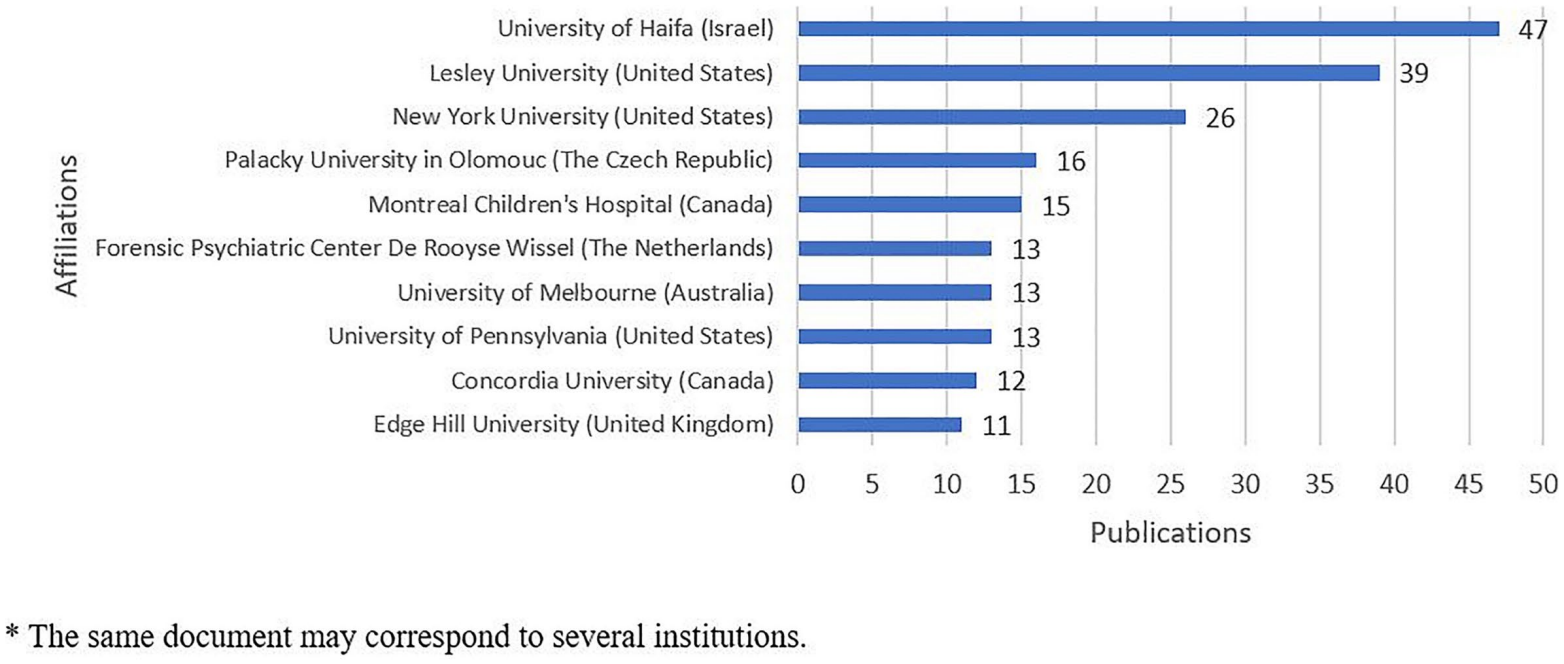
Most relevant affiliation. ^*^The same document may correspond to several institutions.

The most productive institutions were identified in response to the research question (RQ1) on the productivity of research in the field of drama therapy indexed in Scopus. These institutions were defined basing on the affiliation indicated by the authors of the scientific articles and the data were extracted using the Biblioshiny application. This data was then refined and visualized using MS Excel. The authors of the study dataset indicated their affiliation to 265 different institutions. However, 47 (11.9%) publications did not specify the authors’ affiliation. The results show that 162 (61%) institutions were mentioned only once. The most frequently mentioned institutions were Haifa University from Israel with 47 publications, Lesley University with 39 publications and New York University with 26 publications, both from the United States. Figure 6 shows a graph showing the cases where the authors’ affiliation is mentioned more than 10 times.

### Countries

In response to research question RQ1 on the productivity of research in drama therapy indexed in Scopus, the most productive countries were identified. Using Biblioshiny, the authors’ country affiliations were analyzed and the data were refined and visualized in MS Excel. The results showed that the authors represented 38 countries, of which 8 countries had more than 10 publications. The highest number of publications, 128 (32.41%), was published by United States authors, followed by United Kingdom and Israeli authors with 70 (17.72%) and 42 (10.63%) publications respectively, ranking second and third.

**Figure 7:**
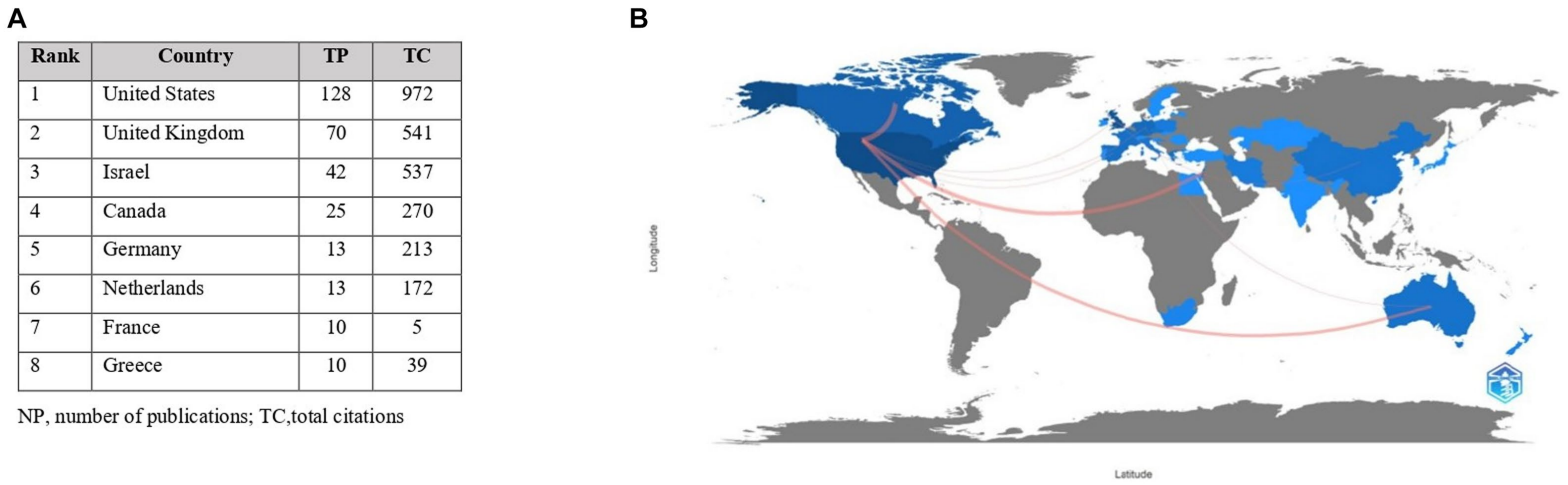
Most influential countries in drama therapy studies. **(A)** Countries with at least ten publicationsin drama. **(B)** Countries’ Collaboration World Map.

In response to research question RQ2 on country impact, the United States was ranked as the leading country in drama therapy research, not only in terms of publications but also in terms of citations. Figure 7A shows the results for the number of publications and citations for countries with at least 10 publications in drama therapy by the authors represented.

To answer research question RQ3 on the countries that have collaborated most often using Biblioshiny, a map of country collaborations was created. Figure 7B shows which countries’ authors have collaborated most frequently on studies indexed in Scopus. Countries with darker colored territories on the map have published more than countries with lighter colored territories. Countries that have not published in the field of drama therapy are shaded in grey. The lines indicate cooperation between countries, the thicker the line, the closer the cooperation. The most productive collaborations were between the United States and Canada (*n* = 7), United States and Israel (*n* = 5), United States and Australia (*n* = 4). There were also two collaborations between United States and German (*n* = 2), Spanish and United Kingdom (*n* = 2) authors, and United Kingdom authors with Chinese (*n* = 2) and Dutch (*n* = 2) authors.

### Publications

To answer the research question (RQ2) on the most popular scientific publications on drama therapy, a citation analysis was performed based on the number of citations received by the publications. Biblioshiny and MS Excel were used for the citation analysis. Table 4 summarizes the 10 publications with the highest total number of citations, the 10 publications with the highest number of citations excluding self-citations, and the 10 publications with the highest number of citations based on the year of publication. Some publications overlapped in this list, so 16 publications were included, in chronological order of year of publication. The 10 most highly results in each category are in bold. The most highly cited of the three criteria is the 2018 Behavioral Sciences systematic review “Creative arts interventions for stress management and prevention - a systematic review” by Martin L., Oepen R., Bauer K., Nottensteiner A., Mergheim K., Gruber H., Koch S.C. This study examines the effectiveness of arts therapy interventions in reducing and preventing stress. It was concluded that the quality of the research is high, but it is worth exploring the specific features of individual art therapy specializations and begin to relate these with different contexts and population groups (Martin et al., 2018).

**Table 4:** Top publications cited.

PY	Publication	Author(s)	Journal	TC	CES	TC/Y
1982	Developmental approaches in drama therapy	Johnson D.R.	The Arts in Psychotherapy	**46**	**36**	1.1
1983	The use of distancing in drama therapy	Landy R.J.	The Arts in Psychotherapy	**56**	**48**	1.37
2006	On dramatic reality and its therapeutic function in drama therapy	Pendzik S.	Arts in Psychotherapy	**58**	**55**	3.22
2006	The treatment of aggression using arts therapies in forensic psychiatry: results of a qualitative inquiry	Smeijsters H., Cleven G.	Arts in Psychotherapy	**58**	**52**	3.22
2007	Classroom drama therapy program for immigrant and refugee adolescents: a pilot study	Rousseau C., Benoit M., Gauthier M.F., Lacroix L., Alain N., Viger Rojas M., Moran A., Bourassa D.	Clinical Child Psychology and Psychiatry	**67**	**55**	3.94
2007	Drama therapy for schizophrenia or schizophrenia-like illnesses	Ruddy R.A., Dent-Brown K.	Cochrane Database of Systematic Reviews	**48**	**46**	2.82
2015	Complementary and alternative therapies for autism spectrum disorder	Brondino N., Fusar-Poli L., Rocchetti M., Provenzani U., Barale F., Politi P.	Evidence-based Complementary and Alternative Medicine	**52**	**43**	**5.78**
2016	Designing personal grief rituals: an analysis of symbolic objects and actions	Sas C., Coman A.	Death Studies	45	32	**5.63**
2017	Critical perspectives in the arts therapies: response/ability across a continuum of practice	Sajnani N., Marxen E., Zarate R.	Arts in Psychotherapy	38	30	**5.43**
2018	A systematic review of the efficacy of creative arts therapies in the treatment of adults with PTSD	Baker F.A., Metcalf O., Varker T., O’Donnell M.	Psychological Trauma: Theory, Research, Practice, and Policy	**47**	**41**	**7.83**
2018	Creative arts interventions for stress management and prevention – a systematic review	Martin L., Oepen R., Bauer K., Nottensteiner A., Mergheim K., Gruber H., Koch S.C.	Behavioral Sciences	**62**	**52**	**10.33**
2019	Creative art therapy for mental illness	Chiang M., Reid-Varley W.B., Fan X.	Psychiatry Research	40	30	**8**
2019	Creative arts interventions to address depression in older adults: a systematic review of outcomes, processes, and mechanisms	Dunphy K., Baker F.A., Dumaresq E., Carroll-Haskins K., Eickholt J., Ercole M., Kaimal G., Meyer K., Sajnani N., Shamir O.Y., Wosch T.	Frontiers in Psychology	**55**	**42**	**11**
2019	Integrative systematic review of drama therapy intervention research	Feniger-Schaal R., Orkibi H.	Psychology of Aesthetics, Creativity, and the Arts	34	19	**6.8**
2021	From therapeutic factors to mechanisms of change in the creative arts therapies: a scoping review	de Witte M., Orkibi H., Zarate R., Karkou V., Sajnani N., Malhotra B., Ho R.T.H., Kaimal G., Baker F.A., Koch S.C.	Frontiers in Psychology	38	22	**12.67**
2022	Participation in life-review playback theater enhances mental health of community-dwelling older adults: a randomized controlled trial	Keisari S., Palgi Y., Yaniv D., Gesser-Edelsburg A.	Psychology of Aesthetics, Creativity, and the Arts	11	5	**5.5**

PY, publication year; TC, total citations; CES, citations exclude self-citations; CES/Y, citations exclude self-citations per year.

To determine the intellectual structure of the field of drama therapy, research question Q4 asked which documents were most frequently cited by drama therapy researchers in studies indexed in the Scopus database. Document co-citation analysis, one of the most used and reliable bibliometric methods, was used to achieve this goal (Zupic and Čater, 2015). The co-citation analysis is based on references to find out which documents the authors of this dataset refer to in their studies. VOSviewer software was used to select author references from this study dataset that were cited at least five times. Of the 14098 cited references, only 14 met this threshold and the strength of the co-citation links with other references was calculated for each reference. The publications were arranged in chronological order according to the year of publication and are shown in Table 5. The content of these publications can identify paradigm shifts and shifts in opinion (Pasadeos et al., 1998). The analysis can be used as a supporting tool by drama therapy students and researchers for the development of reading lists.

**Table 5:** The most popular publications referenced by authors in the field of drama therapy.

PY	Publication	Author(s)	Journal	TC	TLS
1983	The use of distancing in drama therapy	Landy, R.J.	The Arts in Psychotherapy	11	3
1986	Drama therapy: concepts and practices	Landy, R.J.	Springfield	5	2
1996	Drama as therapy: theatre as living	Jones, P.	Routledge	5	2
2003	Therapeutic theatre and well-being	Snow, S., D’Amico, M., Tanguay, D.	Arts in Psychotherapy	5	3
2006	Using thematic analysis in psychology	Braun, V., Clarke, V.	Qualitative Research in Psychology	5	2
2006	On dramatic reality and its therapeutic function in drama therapy	Pendzik, S.	Arts in Psychotherapy	22	9
2006	A review of dramatherapy research in schizophrenia: methodologies and outcomes	Yotis, L.	Psychotherapy Research	6	3
2011	Playback theatre and recovery in mental health: preliminary evidence	Moran, G.S., Alon, U.	Arts in Psychotherapy	5	7
2012	Playing and reality	Winnicott, D.W.	Playing and Reality	5	1
2013	The body politic: the relevance of an intersectional framework for therapeutic performance research in drama therapy	Sajnani, N.	Arts in Psychotherapy	5	1
2014	The effect of drama-based group therapy on aspects of mental illness stigma	Orkibi, H., Bar, N., Eliakim, I.	Arts in Psychotherapy	5	6
2015	Self-revelatory performance: a form of drama therapy and theatre	Emunah, R.	Drama Therapy Review	6	4
2016	Developmental Transformations short form as a stress reduction method for children	R Pitre, C Mayor, DR Johnson	Drama Therapy Review	5	3
2017	Life-crossroads on stage: integrating life review and drama therapy for older adults	Keisari, S., Palgi, Y.	Aging and Mental Health	5	6

PY, publication year; TC, total citations; TLS, total link strength.

### Keywords

**Figure 8:**
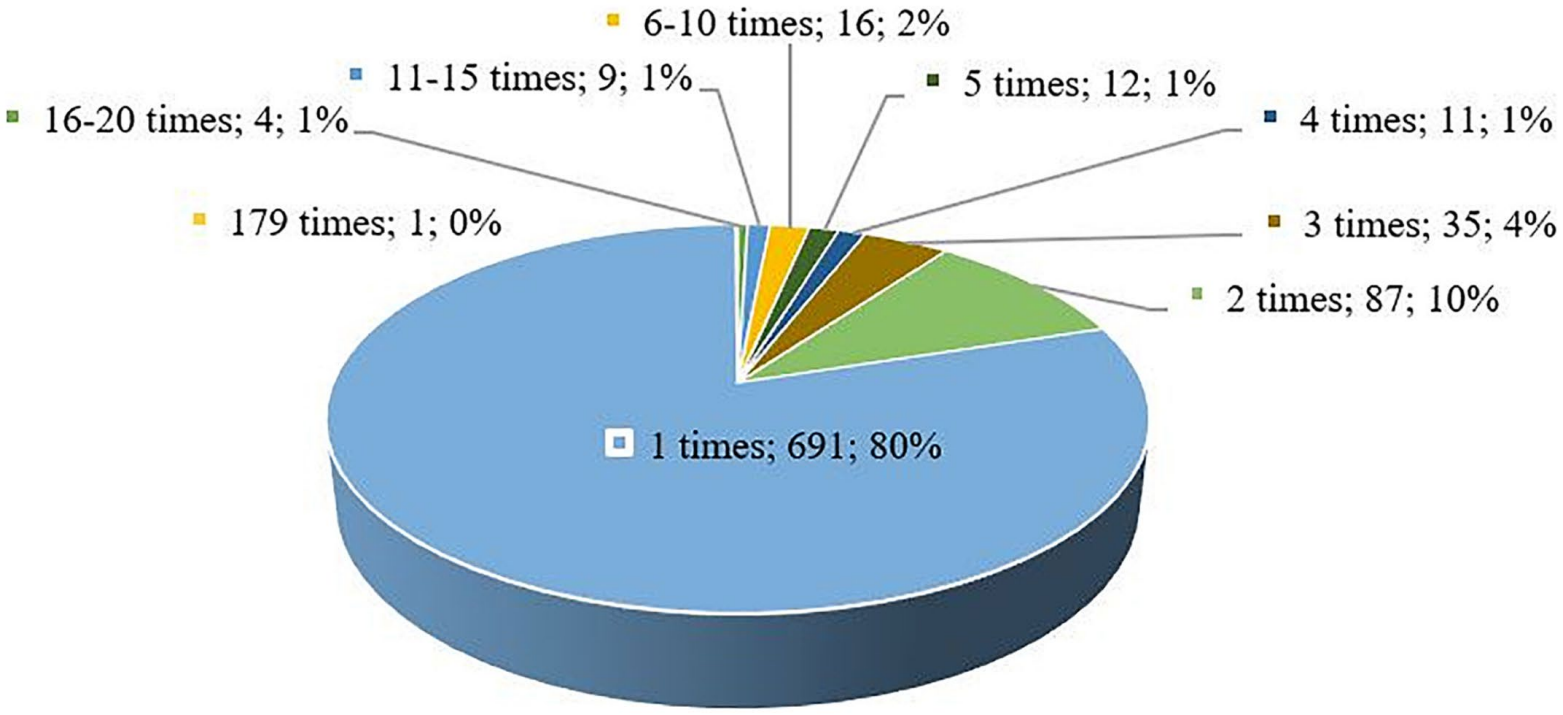
Frequency of occurrence of authors keywords.

To answer the research question (RQ5) about the most frequent keywords and to investigate their thematic interrelationships, we performed a keyword analysis using VOSviewer and MS Excel. A total of 1914 keywords were identified, of which 1195 were indexed keywords and 888 were author keywords. Initial analysis of the category “author keywords” revealed the need for data cleaning. Words in both singular and plural forms were merged if they retained meaning (e.g., ‘disability’ and ‘disabilities’) words with different spellings but the same meaning (e.g., ‘theatre’ and ‘theater’) were merged and words and word compounds with and without hyphens were merged. As a result, 863 keywords were analyzed using the full count method. As shown in Figure 8, 691 (80%) keywords occurred only once, 87 (10%) keywords occurred twice, 35 (4%) keywords occurred three times, and 12 (1%) keywords occurred four times.

The content analysis revealed six dominant research categories. The broadest of them described occupations or fields–drama therapy, art therapy, creative arts therapy, music therapy, dance movement therapy, arts therapy, psychodrama, psychotherapy, group therapy, therapeutic theater, therapy, family therapy. The most common demographic groups studied were adolescents, children, and older adults, but the most studied pathologies or conditions– dementia, mental health, schizophrenia, and trauma. The most frequently mentioned environment was education, school, and prison. Basic processes or methods were embodiment, developmental transformations, drama, role theory, art, performance, and role. Systematic reviews are often mentioned as a type of study.

**Figure 9:**
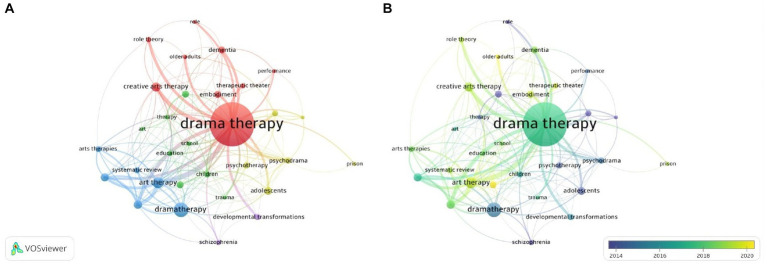
Keyword co-occurrence network of the most frequent keywords. **(A)** Network Visualization. **(B)** Overlay Visualization.

Using VOSViewer software, a cluster analysis was performed to group related keywords into distinct thematic clusters. Figure 9A shows the keywords that appear together six or more times in publications, where the size of the nodes is proportional to their frequency of occurrence. The lines between the keywords show the links between them. The results were divided into five clusters of different colors, which highlighted the thematic relationship of the keywords. The red cluster (cluster 1) contained the following keywords: creative arts therapy, dementia, drama therapy, embodiment, older adults, performance, role, role theory, therapeutic theater. The Green cluster (Cluster 2) summarizes the keywords: art, children, drama, education, mental health, school, therapy, trauma. The blue cluster (cluster 3) contained the following keywords: art therapy, arts therapies, dance movement therapy, drama therapy, music therapy, systematic review. The yellow cluster (cluster 4) contains the keywords: adolescents, family therapy, group therapy, prison, psychotherapy. The purple cluster (cluster 5) consists of developmental transformation, schizophrenia.

Keywords that occurred six or more times are listed in Table 6, which shows their frequency of occurrence and the strength of the links, thematic clusters, and research categories.

**Table 6:** Keywords mentioned at least six times.

Keyword	Occurrences	Total link strength	Cluster	Thematic categories
Drama therapy	179	858	Red (1)	Occupations or fields
Art therapy	20	120	Blue (3)	Occupations or fields
Adolescents	18	87	Yellow (4)	Demographic groups
Creative arts therapy	17	90	Red (1)	Occupations or fields
Music therapy	16	96	Blue (3)	Occupations or fields
Dance movement therapy	14	86	Blue (3)	Occupations or fields
Arts therapy	13	70	Blue (3)	Occupations or fields
Dementia	12	57	Red (1)	Pathologies or conditions treated
Embodiment	12	70	Red (1)	Basic processes or methods
Mental health	12	69	Green (2)	Pathologies or conditions treated
Developmental transformations	11	48	Purple (5)	Basic processes or methods
Drama	11	55	Green (2)	Basic processes or methods
Psychodrama	11	67	Yellow (4)	Occupations or fields
Psychotherapy	11	61	Yellow (4)	Occupations or fields
Children	10	60	Green (2)	Demographic groups
Systematic review	10	50	Blue (3)	Type of study
Role theory	9	45	Red (1)	Basic processes or methods
Schizophrenia	9	38	Purple (5)	Pathologies or conditions treated
Education	8	45	Green (2)	Environment
Group therapy	8	44	Yellow (4)	Occupations or fields
Therapeutic theater	8	45	Red (1)	Occupations or fields
Older adults	7	38	Red (1)	Demographic groups
School	7	40	Green (2)	Environment
Therapy	7	34	Green (2)	Occupations or fields
Art	6	47	Green (2)	Basic processes or methods
Family therapy	6	34	Yellow (4)	Occupations or fields
Performance	6	41	Red (1)	Basic processes or methods
Prison	6	33	Yellow (4)	Environment
Role	6	29	Red (1)	Basic processes or methods
Trauma	6	33	Green (2)	Pathologies or conditions treated

Figure 9B shows the chronological time of appearance of the keywords from blue to yellow. Topics trending around 2014 were schizophrenia adolescents etc. Topical research topics launched around 2018: dementia trauma children etc. The keywords highlighted in yellow indicate currently relevant research topics which are the focus for the post-2020 period. One of the most researched demographic groups today is older adults. This demographic group is the most actively researched by Shoshi Keisari from the University of Haifa in Israel. The author is also conducting research on the hottest topic of the moment - mental health which has also been addressed by Nisha Sajnai of New York University and Jason Scott Frydman of Lesley University in the United States.

## Discussion

Based on data from the Scopus database, this study performed a bibliometric analysis of research trends in the field of drama therapy. It identified publication impact indicators and described the intellectual, social, and conceptual structure of research by analyzing 395 journal research papers published between 1949 and 2022 according to several parameters: authors, institutions, countries, languages, disciplines, journals, citations, references, and keywords.

The results show an increase in the number of publications, with growth dynamics during periods associated with historically important events in the field of drama therapy. They include the launch of journal The Arts in Psychotherapy in 1982, the founding of the European Federation of Drama therapy in 2013, and the launch of the North American Drama Therapy Association (NADTA) journal Drama Therapy Review in 2015 (Sajnani, 2015). This indicates trends towards the academization of the field of drama therapy and supports the claims of previous research (Jones, 2015; Feniger-Schaal and Orkibi, 2020; de Witte et al., 2021; Constien and Junker, 2023).

Both above are specialist journals in the field of art therapy and drama therapy, and within the dataset of this study, they are the leading journals. This is followed by Frontiers in Psychology, the most prominent international journal in the field of psychology, which publishes strictly peer-reviewed research. Nearly half of the publications appeared in these three journals. Frontiers in Psychology has the highest Scopus citation metrics of the above, although it must be said that with the advent of the digital age, the link between journal citation and impact factor is diminishing. In the paper era, the journal was perceived as a whole, whereas now the role of individual articles that can be found in databases or other electronic platforms is increasing (Lozano et al., 2012).

A chronological look at the most frequently cited types of work in the research on drama therapy shows that this field of research has evolved considerably. It has evolved from early studies on the basic and theoretical concepts of drama therapy to studies on therapeutic interventions for different patient groups and systematic reviews. Systematic reviews, as a type of research with a high level of evidence, represent a mature stage in this field of research (Ratnani et al., 2023). Observational studies show that researchers most often refer to systematic reviews, which confirms that these studies significantly facilitate understanding and orientation in the field of drama therapy. In current research, there is a tendency for systematic reviews to summarize the specializations of drama therapy, art therapy, music therapy, dance therapy and movement therapy (Baker et al., 2018; Martin et al., 2018; Chiang et al., 2019; Dunphy et al., 2019), which reflects their inter-professional nature.

The results show that only a minority of authors have multiple publications. This could indicate a trend that most drama therapists do not pursue further research after completing their studies, possibly due to insufficient national support for doctoral programs. It is also possible that authors who have published only one study have focused on drama therapy as a peripheral research topic. This relationship is consistent with the theoretical underpinnings of Lotka’s Law (Lotka, 1926). At the same time, the research interests of leading authors in the field of drama therapy are sometimes only part of a broad spectrum of research areas, including psychodrama research, which was not included in this study. The participation of authors from other professions, including medicine or psychotherapy, in the research is a reflection of the close links between these fields and the interdisciplinary nature of drama therapy. In general, today’s leading influential authors in drama therapy are affiliated as faculty members in leading universities, associations, and journal editorial boards. This is a testimony to the importance of scientific infrastructure in research. The importance of funding for the development of drama therapy research is evident. Public data from the most influential authors show significant private and academic grant support, as well as funding from international programs. The United States has the highest scientific performance and, according to a UNESCO report, ranks first in the world in research funding as a share of gross domestic product (GDP) (Dubashi and Ramadass, 2023). Overall, the most influential countries in drama therapy research are the United States, Israel and the United Kingdom, where the development of drama therapy has its roots from its beginnings.

The contribution of the pioneers of the field is still unsurpassed, their work on the basic concepts of drama therapy is still cited every year and continues to influence the development of the field. The intellectual structure of the field of drama therapy can be revealed by the documents that have been most frequently referred to by the authors in their research on drama therapy. These include works that are not about drama therapy but have influenced the theoretical basis of research, such as psychoanalyst David Winnicott’s “Play and Reality” (Winnicott, 2005). The methodology of thematic analysis used in psychological research is much cited (Braun and Clarke, 2006).

There is a significant trend in the patterns of collaboration between authors, where decades of research in the field of drama therapy show a significant and growing shift from single-authored to team-authored research. The increase in co-authorship is not only in the field of drama therapy (Constien and Junker, 2023), but can also be observed in other fields (Pessin et al., 2023). Alongside this, it is evident that authors tend to collaborate in inter-professional and multidisciplinary teams, in line with the World Health Organization’s call for interdisciplinary collaboration (Fancourt and Finn, 2019). The social fabric of drama therapy research is characterized by a network of authors, institutions and countries that stretches across continents around the world - North America, Asia, Australia, and Europe, however, collaboration between researchers from different countries is not frequent. This could be explained by the different legal frameworks and professional status of drama therapy in different countries around the world.

The study reveals that the conceptual structure of drama therapy is characterized by the frequent co-occurrence of six keywords: drama therapy, art therapy, music therapy, dance and movement therapy, and systematic reviews. The common use of these keywords indicates that the systematic reviews analyze the four specializations of art therapy simultaneously. This could indicate a common conceptual basis for these specializations, as well as a stage in the development of the field when sufficient research has accumulated in each of these specializations to summarize their content. However, 80% of the keywords identified occur only once, indicating the need for a common terminology system for post-drama therapy. Such a system could contribute to conceptual clarity and the effectiveness of scientific communication. As the results of the study show, drama therapy is studied in the fields of medicine, psychology, health care, arts, humanities and social sciences and the terminology differs in each of these fields. Interdisciplinary research cooperation would contribute to the development of a common terminology system. Currently, the most pressing research topics in the field of drama therapy are related to mental health crisis and its manifestations, such as depression, anxiety, suicide rates (Letrondo et al., 2023). Likewise, given the increase in life expectancy (World Health Organization, 2015, 2017), there will continue to be challenges in the future, including an increase in the number of people with dementia (Nichols et al., 2022). These challenges will remain as hot topics in the research focus of the field of drama therapy in the long term (World Health Organization, 2015, 2017). Periodic updates on current research topics could improve scientific communication.

This study has several limitations. Inaccuracies in bibliographic references were found during the data selection and processing process. This made it difficult to perform an accurate analysis using RStudio and VOSviewer. The data were processed, merged, and cleaned, which may have led to minor changes, which are explained in detail in the relevant section of the text. Publication references were not manually cleaned due to their large size, which could have led VOSviewer to identify the same document as two different ones, which could have led to inaccurate results in the co-citation analysis. The keyword analysis did not use the full content of the studies, so it would be useful in the future to analyze the data more thoroughly using, for example, the synthetics knowledge synthesis methodology (Kokol et al., 2022).

Only the Scopus database was used and only one type of document was included, namely articles and reports, excluding other types of scientific literature such as books, book chapters and conference proceedings. However, the study includes a rigorous methodology, using a database that is recognized as reliable among researchers worldwide. At the same time, drama therapy as a method has been successfully applied in other settings and contexts. Its development path points towards theory following practice. It is therefore important to maintain the creativity inherent in the field and not to exclude unindexed studies and unreviewed literature as an irrelevant basis for research. In the future, it would be useful to conduct an altmetrics analysis that also allows for the evaluation of various unpublished studies (Bornmann and Haunschild, 2016). This type of review could also potentially be a solution to the lack of systematization of terminology, contributing to the development of a common conceptual and terminological systematization.

It should be noted that 15 and 25% of all journals, including the United Kingdom journal Dramatherapy, are not indexed in the leading multidisciplinary bibliographic databases Scopus and Web of Science (Constien and Junker, 2023). Moreover, some publications are only available in institutional databases, limiting their accessibility to a wider audience. While these materials are a valuable resource for students and researchers, they do not contribute to the dissemination of drama therapy literature in the scientific community. These limitations create barriers to the uptake of evidence-based practice in healthcare and to the integration of drama therapy in medical and healthcare settings, according to industry professionals (Nussbaumer-Streit et al., 2022). An open science approach would address the challenges of access to publications in drama therapy research by allowing researchers to share and analyze research data in a trusted environment that is not constrained by technology or divided by institutional boundaries.

Further research progress in the field of drama therapy would be facilitated by analyzing funding models using data provided to the databases by funding agencies, for example following an approach already developed (Kokol and Blažun Vošner, 2018). This would allow identifying which topics receive the most significant financial support. With the increasing pace of scientific development, some of the results of bibliometric analysis could soon become obsolete. It is therefore recommended that future studies be complemented by bibliographic coupling analysis to provide an up-to-date research base. This method will allow the mapping of publications up to 5 years old and the identification of current research topics, as well as the identification of groups of authors collaborating in this scientific field (Zupic and Čater, 2015). In the future bibliometric analysis could become an equivalent approach to systematic reviews, complemented by altmetric studies.

## Conclusion

To the best of our knowledge, this is the third study on research in the field of drama therapy using bibliometric analysis, but first study to use the Scopus database and Biblioshiny and VOSviewer software. Unlike the previous studies, this one is conducted in a broader context, based on a of publications without language, time and geographical limitations. The novelty of the study is the up-to-date results of the scientific performance in the field of drama therapy, complemented by citation analysis and science mapping. The results of the analysis reflect the evolution of the field from its historical roots to its academic maturity, highlighting its current dynamic growth and the trend of drama therapy to establish itself as an interdisciplinary field in the healthcare system.

The results of the study can be useful for the drama therapy research community by identifying research gaps, relevant research topics and helping to select journals for publication. This analysis can serve as a starting point or as a complementary method for other studies. The results of the study can serve drama therapy professionals to navigate research to inform their practice in scientific research. They can also be useful for students to simplify their literature search and selection. The results of this work are also useful for educational librarians in facilitating and justifying subscription choices and purchasing processes. The results of this work may be useful to those in other fields who wish to gain a broader understanding of research trends in drama therapy.

## Data availability statement

The data analyzed in this study is subject to the following licenses/restrictions: The datasets generated for this study are available on request to the corresponding author. Requests to access these datasets should be directed to ŽK, zanetekorde@gmail.com.

## Author contributions

ŽK: Data curation, Formal analysis, Investigation, Methodology, Software, Visualization, Writing – original draft, Conceptualization, Resources. SŠ: Conceptualization, Methodology, Project administration, Resources, Supervision, Validation, Writing – review & editing. KM: Conceptualization, Methodology, Project administration, Resources, Supervision, Validation, Writing – review & editing.
